# Association between the Triglyceride Glucose Index and Hyperuricemia in Patients with Primary Hypertension: A Cross-Sectional Study

**DOI:** 10.1155/2023/5582306

**Published:** 2023-06-15

**Authors:** Shanshan Liu, Zhixian Zhou, Miao Wu, Hao Zhang, Yao Xiao

**Affiliations:** ^1^Department of Cardiology, Affiliated Hospital of Jiangxi University of Traditional Chinese Medicine, Nanchang, Jiangxi, China; ^2^Jiangxi University of Traditional Chinese Medicine, Nanchang, Jiangxi, China

## Abstract

**Objective:**

The aim of this study was to investigate the association between the triglyceride glucose (TyG) index and hyperuricemia (HUA) in patients with grades 1–3 hypertension. *Study Design*. This is a cross-sectional study. A total of 1,707 patients from the cardiovascular department of Affiliated Hospital of Jiangxi University of Traditional Chinese Medicine were studied. In this study, 899 patients with grades 1-2 hypertension were included, of which 151 had HUA; additionally, 808 patients with grade 3 hypertension were included, of which 162 patients had HUA. This study obtained all patient data from the electronic medical record system of the Affiliated Hospital of Jiangxi University of Traditional Chinese Medicine. The TyG index was calculated as Ln (triglycerides × fasting glucose/2). Hyperuricemia was defined as uric acid ≥420 *μ*mol/L (7 mg/dL). Multivariate logistic regression, penalized spline regression, and generalized additive models were used to evaluate the association between the TyG index and HUA. Stratified analyses were performed to assess the association in populations with different grades of hypertension.

**Results:**

The average TyG index was 8.71 ± 0.58. After adjusting for correlated variables, the logistic regression analysis revealed a positive correlation between the TyG index and HUA (OR = 1.83; 95% CI: 1.40–2.39). Smooth curve fitting showed that this correlation was linear in the whole range of the TyG index. In the subgroup analysis, the TyG index more strongly associated with HUA in the grades 1-2 hypertension group (OR = 2.22; 95% CI: 1.44–3.42) compared to that in the grade 3 hypertension group (OR = 1.58; 95% CI: 1.11–2.24; *P* for interaction = 0.03). In addition, this association was consistent in all models.

**Conclusion:**

The TyG index was positively associated with HUA in patients with hypertension, and the association was more strongly confirmed in those with grades 1-2 hypertension rather than in those with grade 3 hypertension.

## 1. Introduction

Hyperuricemia (HUA) is a metabolic syndrome caused by a purine metabolism disorder. According to the Chinese guidelines [1], HUA can be diagnosed regardless of sex when serum uric acid (SUA) is ≥420 *μ*mmol/L (7 mg/dL). Hyperuricemia can directly cause gout and uric acid nephropathy through sodium urate crystals' deposition. Meanwhile, HUA is an independent risk factor for chronic kidney disease, cardiovascular and cerebrovascular diseases, diabetes, and other cardiovascular events [2–4]. Regarding its pathogenic mechanism, a series of studies have shown that HUA is closely related to insulin resistance (IR) [5–7].

Insulin resistance is a clinical and biochemical disorder that can lead to impaired glucose tolerance and further cause diabetes. Previous studies have proved that IR is associated with obesity, cardiovascular diseases, hypertension, and other diseases of impaired insulin sensitivity and metabolic syndrome [8]. The triglyceride glucose (TyG) index is a cheap, simple, and reliable substitute for IR compared to the homeostasis model (HOMA-IR) index [9]. Thus, the TyG index can be used as an early diagnostic indicator of IR and is helpful in identifying the population at a risk of cerebrocardiovascular disease [10–13].

Several previous studies have proved that the TyG index is associated with HUA [14–16]. However, only a few studies have analyzed the association between the TyG index and HUA in patients with hypertension [16, 17]. Previous studies have focused on all populations with hypertension, with few studies investigating different hypertension grades. Therefore, our study concentrated on the association between the TyG index and HUA in the specific population.

## 2. Methods

### 2.1. Participants

**Figure 1:**
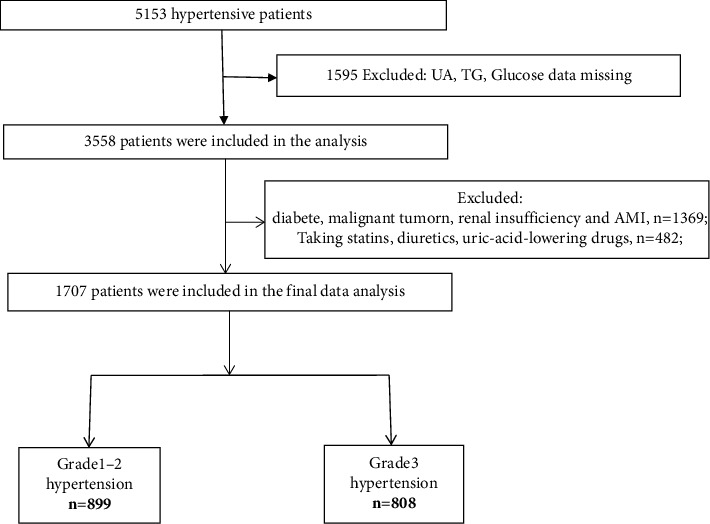
Flowchart of participants' selection. UA, uric acid; TG, triglyceride; AMI, acute myocardial infarction.

Our study included a total of 5,153 in-patients with primary hypertension from January, 2020, to December, 2021, who attended the Affiliated Hospital of Jiangxi University of Traditional Chinese Medicine. All participants were adults (aged 18 years or over) and hospitalized in the Department of Cardiology. Primary hypertension was diagnosed by referring to the 2018 ESC/ESH [18] guidelines for the management of arterial hypertension, which defines hypertension as office systolic blood pressure ≥140 mmHg and/or diastolic blood pressure ≥90 mmHg, when blood pressure value are measured in a resting and sitting position. Final blood pressure values were obtained by averaging three consecutive measurements, following a protocol that required at least 5 minutes of seated rest and two repeated measurements with 5-minute intervals. In-patients who were taking antihypertensive medications or self-reported hypertensive diagnosis were also included. The exclusion criteria were as follows: (1) those with incomplete SUA, triglyceride (TG), and glucose data; (2) those with secondary hypertension, diabetes, acute myocardial infarction, renal insufficiency (estimated glomerular filtration rate (eGFR) <60 mL/min per 1.73 m^2^), or malignant tumor; (3) those taking lipid-lowering drugs in the past 1 month; and (4) those with a history of using diuretics and other drugs that may affect the metabolism of uric acid within 2 months of inclusion. Finally, a total of 1,707 consecutive patients with grades 1–3 primary hypertension were included in our study, of which 899 had grades 1-2 hypertension and 808 patients had grade 3 hypertension.  Figure 1 describes the initial sample population and exclusion criteria. Ethics approval was obtained from the Ethics Committee of the Affiliated Hospital of Jiangxi University of Traditional Chinese Medicine (no.: JZFYLL20220727034). The data were anonymous; thus, the requirement for informed consent was waived.

### 2.2. Data Collection and Definition

Clinical data, such as sex, age, medical history, as well as drug and blood test data, were collected from the electronic medical record system. To obtain blood samples, patients fasted overnight for 10–12 h, and then 4-5 mL venous blood was drawn the next morning. All samples were tested in the Department of Laboratory Medicine, Affiliated Hospital of Jiangxi University of Traditional Chinese Medicine. The following lab methods were used: fast blood glucose was assessed using the hexokinase method; SUA and blood urea nitrogen (BUN) were measured using the enzyme method; blood creatinine (Scr) was measured using the picric acid method; TG and total cholesterol (TC) were tested using the oxidase method; biochemical indicators, such as lipoprotein a (LPa), high density lipoprotein cholesterol (HDL-C), low density lipoprotein cholesterol (LDL-C), d-dimer, and high-sensitivity C-reactive protein (hs-CRP), were measured using immunoturbidimetry; the international normalized ratio (INR) was calculated using its formula; serum albumin (ALB) was analyzed using the bromide green method; alanine aminotransferase (ALT) and aspartate aminotransferase (AST) were assessed using the enzymatic rate method; and all of the previous biomarkers were analyzed using an automatic biochemical analyzer (Siemens advia2400). The TyG index = Ln (TG [mg/dL] × fasting glucose [mg/dL]/2) [19]. The modified modification of diet in renal disease (MDRD) equation was used to estimate eGFR [20]. Hyperuricemia was diagnosed as SUA ≥ 420 *μ*mol/L in men and women using the 2019 Chinese guidelines for the diagnosis and treatment of HUA and gout [1].

### 2.3. Statistical Analysis

Continuous variables were presented as the mean ± standard deviation or as the median (interquartile range), and categorical variables were presented as absolute values and/or frequency (%). The baseline characteristics among the groups organized according to grades of hypertension (grades 1-2 or grade 3) were compared using chi-square tests (categorical variables), one-way analysis of variance (normal distribution), and the Kruskal–Wallis (skewed distribution) tests, respectively.

Multivariate logistic regression analyses were performed to assess the independent association between the TyG index and HUA after adjusting for correlated variables in the three models. The variables were selected based on the following: clinical importance, statistical significance in the univariable analyses, and an estimated variable change of at least 10% of the potential confounding effects. The restricted cubic spline model (a fitted smooth curve) was used to determine the dose-response relationship of the TyG index with SUA and HUA.

Subgroup analyses were stratified by relevant effect covariates as follows: age (<65 years *vs*. ≥65 years), hypertension grades (1-2 *vs*.3), sex (male *vs*. female), TyG index (tertile 1, 7.1–8.4; tertile 2, 8.4–8.9; and tertile 3, 8.9–11.8), and TG (<1.7 mmol/L *vs*. ≥1.7 mmol/L). In the subgroup analyses of hypertension grades, we used the generalized additive model to analyze the dose-response association among the TyG index, SUA, and HUA. In the three models, pertinent covariables included age, sex, ALB, ALT, AST, Scr, BUN, d-dimer, INR, eGFR, hypertension grade, LDL-C, HDL-C, and LPa. All analyses were performed using R Statistical Software (https://www.R-project.org, The R Foundation) and Free Statistics software versions 1.7. A two-tailed *P* value <0.05 was considered to be statistically significant for all analyses.

## 3. Results

### 3.1. Baseline Characteristics

Of the 1,707 patients, the mean age was 62.97 ± 12.87 years and 786 patients were men. In the grades' 1-2 hypertension group, 403 (44.7%) patients were men. In the grade 3 hypertension group, 384 (47.5%) patients were men. The average TyG index was 8.71 ± 0.58, and there was no significant difference between the two groups. Of the 1,707 patients, 313 (18.3%) had HUA, including 151 (16.8%) with grades 1-2 hypertension and 162 (20%) with grade 3 hypertension. Missing data for ALB (0.1%), d-dimer (4.0%), and INR (4.0%) were imputed using the mean value. In the grades' 1-2 hypertension group, the levels of SUA, glucose, and hs-CRP were lower than those in the grade 3 hypertension group (all *P* < 0.05). There was no significant difference in the age and sex of the participants between the groups (*P* > 0.05, Table 1). In addition, there was no significant difference in the value of ALB, LDL-C, HDL-C, AST, Scr, BUN, TC, INR, eGFR, ALT, homocysteine (HCY), LPa, d-dimer, and TG between the two groups (*P* > 0.05, Table 1).

### 3.2. The Positive Association of the TyG Index with HUA

As shown in Table 2, the univariate analysis revealed that the TyG index, sex, age, ALB, HDL-C, AST, Scr, BUN, glucose, eGFR, ALT, LPa, and TG were associated with HUA in patients with primary hypertension (all *P* < 0.05). Furthermore, the multivariate analysis also revealed that the TyG index was positively associated with SUA and HUA after adjusting for potential confounding factors, as demonstrated in Tables 2 and 3.

**Figure 2:**
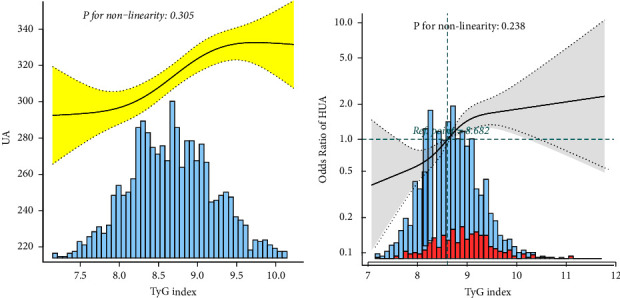
Dose-response association between the TyG index and the risk of HUA. (a) TyG index and SUA and (b) TyG index and HUA. All adjusted for age, sex, ALB, ALT, AST, Scr, BUN, d-dimer, INR, eGFR, hypertension grades, LDL-C, HDL-C, and LPa. In this figure, we can find the association displayed a linear pattern in the whole range of TyG. TyG, triglyceride-glucose index; HUA, hyperuricemia; SUA, serum uric acid; ALB, serum albumin; ALT, alanine aminotransferase; AST, aspartate aminotransferase; Scr, blood creatinine; BUN, blood urea nitrogen; INR, international normalized ratio; eGFR, estimated glomerular filtration rate; LDL-C, low-density lipoprotein cholesterol; HDL-C, high-density lipoprotein cholesterol; and LPa, lipoprotein a.

Figure 2 shows the dose-response relationship among the TyG index, SUA, and HUA levels in patients with primary hypertension. We found a linear relationship between the TyG index and HUA in patients with primary hypertension and an increasing trend in the incidence of HUA with the increasing TyG index. When the TyG index was assessed as tertiles, the incidence risks of HUA in the 2^nd^ and 3^rd^ tertiles were 1.45 (95% CI: 0.98–2.15) and 2.40 (95% CI: 1.60–3.60) times greater than that in the lowest tertile (*P* for trend <0.001), as shown in Table 4.

### 3.3. Subgroup Analysis

**Figure 3:**
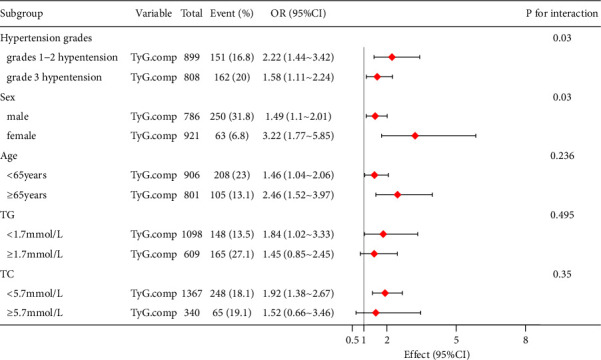
Subgroup analysis of the association between the TyG index and HUA. Adjusted for age, sex, ALB, ALT, AST, Scr, BUN, d-dimer, INR, eGFR, hypertension, LDL-C, HDL-C, and LPa. TyG, triglyceride-glucose index; HUA, hyperuricemia; ALB, serum albumin; ALT, alanine aminotransferase; AST, aspartate aminotransferase; Scr, blood creatinine; BUN, blood urea nitrogen; INR, international normalized ratio; eGFR, estimated glomerular filtration rate; LDL-C, low-density lipoprotein cholesterol; HDL-C, high-density lipoprotein cholesterol; and LPa, lipoprotein a.

To further reveal the association between the TyG index and HUA in different subgroups, we conducted stratified analyses (Figure 3). Some subgroups had no significant interactions: age (<65 *vs*. ≥65 years), TG (<1.7 *vs*. ≥1.7 mmol/L), and TC (<5.7 *vs*. ≥5.7 mmol/L). However, we found significant interactions in two subgroups: sex (male *vs*. female) and hypertension grade (grades 1-2 *vs*. grade 3). Women had a greater risk (OR = 3.2, 95% CI: 1.77–5.85) of developing HUA than men (OR = 1.49, 95% CI: 1.1–2.01; *P* for interaction = 0.03). Similarly, the association was strongly confirmed in the grades' 1-2 hypertension group compared to the grade 3 hypertension group (grades 1-2 hypertension: OR = 2.22, 95% CI: 1.44–3.42; grade 3 hypertension: OR = 1.58, 95% CI: 1.11–2.24; *P* for interaction = 0.03). After the TyG index was divided into tertiles, the risks of HUA in the 2^nd^ and 3^rd^ tertiles were 2.57 (95% CI: 1.40–4.71) and 3.02 (95% CI: 1.57–5.82) times higher than that in the lowest tertile in the grades' 1-2 hypertension group (*P* for trend = 0.002). The risks of HUA in the 3^rd^ tertiles were 1.99 (95%CI: 1.17–3.39) times higher than that in the lowest tertile in grade 3 hypertension group (*P* for trend = 0.004). There was no significant difference between the 2^nd^ tertiles and the 1^st^ tertiles. In the 1^st^–3^rd^ tertiles, the TyG index had a strong significant association with HUA in the grades' 1-2 hypertension group compared to that in the grade 3 hypertension group (*P* for interaction <0.05), as shown in Table 5. The results of the analysis of the association between the TyG index and SUA were consistent and are presented in Table 6.

## 4. Discussion

We analyzed the correlation between the TyG index and HUA in patients with grades 1-3 hypertension. The results showed that the TyG index was positively associated with SUA and HUA. The restricted cubic spline indicated that the association was linear across the TyG range. The risk of HUA gradually increased with an increase in the TyG index. Moreover, the subgroup analyses suggested that the positive association seemed to be strong among grades 1-2 hypertension and women (*P* for interaction = 0.03).

In recent years, there are some studies that have drawn conclusions on the association among the TyG index, SUA, and HUA. Kahaer et al. [21] found that the TyG index had a significant association with HUA and could be used as a risk screening indicator of HUA in the Xinjiang population in China. Meanwhile, da Silva et al. [11] conducted a linear relationship analysis between the TyG index and HUA in the general Chinese population by using the TyG index values to predict HUA. The results are helpful in providing a simple and cost-effective method for the prevention and control of HUA. Furthermore, Zhou et al. [13] included 13,060 hypertensive patients in a cross-sectional study to analyze the association between the TyG index and HUA. The research divided the TyG index into four quartiles and found that the risk of HUA in the highest tertile was 2.79 times higher than that of the lowest tertile, again confirming a strong linear relationship between the TyG index and HUA in a hypertensive population. Our study revealed that when the TyG index was divided into three tertiles, and the risk of HUA was similar to the previous results. We further conducted subgroup analyses for grades 1-2 hypertension and grade 3 hypertension after performing a stratified analysis of the TyG index. The results showed that the HUA risk odds ratio (OR) and regression coefficient of grades 1-2 hypertension were higher than those of grade 3 hypertension (*P* value for interaction <0.05). The difference was consistent in all models, indicating that the difference was steady for these risk factors; thus, the study concluded that the TyG index was positively associated with HUA, and the association was more strongly confirmed in those with grades 1-2 hypertension rather than in those with grade 3 hypertension. However, the mechanisms underlying these differences are not yet fully understood.

Previous studies have confirmed that the TyG index identified HUA differently in women and men [17]. Similarly, our study found that the OR and regression coefficient values of the TyG index were higher in women, suggesting that the TyG index had a strong association with HUA in female populations with hypertension. This may be explained by estrogen being related to complex endocrine factors as well as being a uric acid producing agent. Estrogen also accounts for some differences in lipid metabolism between women and men.

Although our study and previous research have confirmed the association between the TyG index and HUA, the specific mechanisms remain unclear. The most commonly recognized mechanism is related to IR. Previous epidemiological studies have revealed a significant relationship between IR and SUA [22, 23]. After IR, compensatory hyperinsulinemia occurs, leading to decreased uric acid excretion through renal tubular sodium reabsorption, which further causes HUA. In addition, one study proved that early *β*-cell dysfunction was mainly associated with elevated uric acid levels [24]. Insulin resistance also causes HUA through several other pathways, including by inducing systemic inflammation, causing kidney damage, decreasing renal uric acid excretion, and affecting lipid metabolism [25]. Conversely, HUA can lead to IR and inflammation by affecting adipocytes and reducing mitochondrial oxidative stress and nitric oxide bioavailability [26]. HUA and IR are both important risk factors for hypertension. The specific mechanisms are as follows: (1) human studies have shown that HUA may be related to impaired endothelial function [27, 28], (2) animal studies shown that HUA can activate the renin-angiotensin system, which leads to increased systemic BP and vascular resistance [29], and (3) activating the immune system and its related hemodynamic effects [30]. These changes can aggravate systemic hypertension and lead to end-organ damage. Wang et al. [31] conducted a prospective cohort study included 21,999 subjects without hypertension or gout at the baseline. The results showed that the elevated SUA is associated with an increased risk of hypertension, and IR may play an intermediary role in the relationship between SUA and hypertension.

Studies have shown that the TyG index is a more sensitive and specific indicator for IR detection compared to other IR indicators [32]. In addition, some researchers found an association between the TyG index and HUA in populations from different regions.

Our study aimed to explore the association of the TyG index and HUA in hospitalized patients with grades 1–3 hypertension. One main strength of our research was that we conducted stratified analyses in populations with grades 1-2 hypertension and grade 3 hypertension. These results are helpful to show the association between the TyG index and HUA in these specified populations.

Our study had some limitations. First, this was a cross-sectional study, and therefore, the association between the TyG index and HUA in patients with grades 1–3 hypertension is insufficient to draw causal conclusions. In the future, prospective randomized studies could help obtain more evidence to support the associations found in our study. Second, we included only a subset of hospitalized patients in Jiangxi, China, which was not representative of the entire population. In addition, most in-patients had grade 2 and grade 3 hypertension and fewer in-patients had grade 1 hypertension. Therefore, we combined the grade 1 and grade 2 hypertension cases for further analysis. The target population of our study was hospitalized hypertensive patients, most of whom had grades 2-3 hypertension; thus, our study is consistent with clinical reality. Third, data derived from the electronic medical record system lacked information on waist circumference (WC), height, weight, alcohol consumption, and smoking history. These unrecorded risk factors for HUA could be residual confounding variables, potentially causing further bias in our results. However, most studies found that the positive association between the TyG index and HUA was still significant after adjusting for these risk factors [16, 17, 21], and therefore, we consider our conclusions to be reliable.

## 5. Conclusion

In conclusion, our study confirmed the positive association between the TyG index and the risk of HUA in hospitalized patients with hypertension. The association was more strongly confirmed in women than in men and in grades 1-2 hypertension than in grade 3 hypertension.

## Figures and Tables

**Table 1:** Baseline characteristics of the study participants with different grades of hypertension.

	Total (*n* = 1707)	Grades 1-2 hypertension (*n* = 899)	Grade 3 hypertension (*n* = 808)	*P* value
TyG	8.71 ± 0.58	8.67 ± 0.56	8.74 ± 0.60	**0.02**
Sex, *n* (%)				0.25
Male	786 (46.0)	402 (44.7)	384 (47.5)	
Female	921 (54.0)	497 (55.3)	424 (52.5)	0.25
Age, years	62.97 ± 12.87	62.78 ± 12.50	63.17 ± 13.28	0.53
Age groups, *n* (%)				0.21
<65 years	906 (53.1)	490 (54.5)	416 (51.5)	
≥65 years	801 (46.9)	409 (45.5)	392 (48.5)	
ALB, g/L	43.76 ± 3.92	43.87 ± 3.78	43.62 ± 4.07	0.19
LDL-C, mmol/L	2.58 ± 0.88	2.56 ± 0.87	2.59 ± 0.89	0.51
HDL-C, mmol/L	1.48 ± 0.40	1.50 ± 0.40	1.46 ± 0.41	0.06
AST, U/L	22.56 ± 10.84	22.34 ± 9.72	22.81 ± 11.97	0.38
Scr, *μ*mol/L	79.63 ± 18.16	79.38 ± 17.97	79.90 ± 18.39	0.56
BNU, mmol/L	5.22 ± 1.42	5.17 ± 1.33	5.27 ± 1.50	0.17
UA, *μ*mol/L	343.43 ± 83.93	335.95 ± 84.07	351.76 ± 83.03	**<0.001**
Glucose, mmol/L	5.31 ± 0.96	5.25 ± 0.72	5.38 ± 1.17	**0.004**
TC, mmol/L	4.83 ± 1.06	4.80 ± 1.05	4.85 ± 1.07	0.37
INR	1.00 ± 0.11	1.00 ± 0.10	1.01 ± 0.12	0.42
eGFR, mL/min per 1.73 m^2^	90.17 ± 31.15	89.88 ± 24.96	90.49 ± 36.84	0.68
HUA, *n* (%)	313 (18.3)	151 (16.8)	162 (20)	0.08
hs-CRP, mg/L	2.1 (1.7, 3.1)	2.1 (1.7, 2.9)	2.2 (1.8, 3.3)	**0.01**
ALT, U/L	16.3 (11.9, 24.1)	16.2 (11.9, 24.1)	16.5 (11.8, 24.1)	0.48
HCY, *μ*mol/L	9.1 (6.0, 12.3)	9.0 (6.0, 12.2)	9.4 (6.0, 13.0)	0.14
LPa, mg/dL	76.8 (37.2, 172.9)	79.9 (38.0, 175.3)	72.8 (36.0, 171.5)	0.28
D-dimer, mg/L	0.3 (0.2, 0.5)	0.3 (0.2, 0.5)	0.3 (0.2, 0.5)	0.51
TG, mmol/L	1.4 (1.0, 2.0)	1.4 (1.0, 1.9)	1.4 (1.0, 2.0)	0.07

Data are presented as the mean ± SD or median (interquartile range) for skewed variables or as numbers (%) for categorical variables. TyG: triglyceride-glucose index; ALB: serum albumin; LDL-C: low-density lipoprotein cholesterol; HDL-C: high-density lipoprotein cholesterol; AST: aspartate aminotransferase; Scr: blood creatinine; BUN: blood urea nitrogen; UA: uric acid; TC: total cholesterol; INR: international normalized ratio; eGFR: estimated glomerular filtration rate; HUA: hyperuricemia; hs-CRP: high-sensitivity C-reactive protein; ALT: alanine aminotransferase; HCY: homocysteine; LPa: lipoprotein a; and TG: triglyceride. Statistically significant *P*-values in bold for a better follow-up of the results. The bold values mean that the difference between the two groups is significant.

**Table 2:** Results of univariate and multivariate analyses between HUA and all parameters.

Variable	Univariate analysis		Multivariate analysis	
	OR (95% CI)	*P* value	OR (95% CI)	*P* value
TyG	2.47 (1.99–3.07)	**<0.001**	1.83 (1.40–2.39)	**<0.001**
Hypertension grades (1-2 *vs.*3)	1.24 (0.97–1.59)	0.08	1.13 (0.86–1.48)	0.40
Sex (male *vs*. female)	0.16 (0.12–0.21)	**<0.001**	0.24 (0.13–0.45)	**<0.001**
Age, years	0.97 (0.96–0.98)	**<0.001**	0.99 (0.98–1.01)	0.25
ALB, g/L	1.08 (1.04–1.12)	**<0.001**	1.05 (1.01–1.10)	**0.03**
hs-CRP, mg/L	0.99 (0.97–1.01)	0.36		
LDL-C, mmol/L	0.90 (0.78–1.03)	0.13	0.94 (0.79–1.11)	0.44
TG, mmol/L	1.42 (1.28–1.57)	**<0.001**		
HDL-C, mmol/L	0.27 (0.19–0.39)	**<0.001**	0.77 (0.49–1.23)	0.28
ALT, U/L	1.02 (1.02–1.03)	**<0.001**	1.01 (1.000–1.02)	0.20
AST, U/L	1.03 (1.02–1.04)	**<0.001**	1.01 (1.00–1.03)	0.08
Scr, *μ*mol/L	1.04 (1.03–1.05)	**<0.001**	1.01 (0.97–1.04)	0.73
BUN, mmol/L	1.22 (1.12–1.33)	**<0.001**	1.21 (1.09–1.33)	**<0.001**
Glucose, mmol/L	1.13 (1.01–1.27)	**0.03**		
HCY, *μ*mol/L	1.02 (1.00–1.03)	**0.004**		
LPa, mg/dL	0.999 (0.998–0.999)	**0.001**	1.00 (0.999–1.00)	0.29
TC, mmol/L	1.01 (0.90–1.11)	0.92		
D-dimer, mg/L	0.80 (0.61–1.04)	0.09	1.02 (0.80–1.30)	0.90
INR	0.77 (0.22–2.66)	0.68	0.85 (0.22–3.20)	0.81
eGFR, mL/min per 1.73 m^2^	0.99 (0.98–0.99)	**<0.001**	0.99 (0.97–1.02)	0.57

HUA, hyperuricemia; TyG, triglyceride-glucose index; ALB, serum albumin; hs-CRP, high-sensitivity C-reactive protein; LDL-C, low-density lipoprotein cholesterol; TG, triglyceride; HDL-C, high-density lipoprotein cholesterol; ALT, alanine aminotransferase; AST, aspartate aminotransferase; Scr, blood creatinine; BUN, blood urea nitrogen; HCY, homocysteine; LPa, lipoprotein a; TC, total cholesterol; INR, international normalized ratio; eGFR, estimated glomerular filtration rate; OR, odds ratio; and CI, confidence interval. Statistically significant *P*-values in bold for a better follow-up of the results. The bold values mean that TyG index, sex, age, ALB, HDL-C, AST, Scr, BUN, glucose, eGFR, ALT, LPa, and TG were associated with HUA in patients with primary hypertension (all *P* < 0.05). Furthermore, the multivariate analysis also revealed that the TyG index was positively associated with HUA after adjusting for potential confounding factors.

**Table 3:** Results of the univariate and multivariate linear regression between SUA and all parameters.

Variables	Univariate linear regression		Multivariate linear regression	
	*β* coefficient (95% CI)	*P* value	*β* coefficient (95% CI)	*P* value
TyG index	38.61 (31.98–45.23)	**<0.001**	20.99 (14.19–27.8)	**<0.001**
Sex (female *vs.* male)	−77.72 (−84.81–−70.63)	**<0.001**	−50.14 (−59.77–−40.52)	**<0.001**
Age, years	−1.47 (−1.77–−1.17)	**<0.001**	−0.35 (−0.67–−0.03)	**0.03**
ALB, g/L	3.32 (2.32–4.32)	**<0.001**	2.09 (1.05–3.13)	**<0.001**
hs-CRP, mg/L	−0.52 (−1.02–−0.02)	**0.04**		
LDL-C, mmol/L	−2.96 (−7.5–1.57)	0.2	−1.79 (−5.82–2.25)	0.39
TG, mmol/L	16.38 (13.15–19.6)	**<0.001**		
HDL-C, mmol/L	−50.73 (−60.3–−41.15)	**<0.001**	−9.53 (−19.93–0.87)	0.07
ALT, U/L	1.06 (0.84–1.28)	**<0.001**	0.18 (−0.11–0.47)	0.22
AST, U/L	1.23 (0.87–1.6)	**<0.001**	0.55 (0.09–1.01)	**0.02**
Scr, *μ*mol/L	1.77 (1.57–1.97)	**<0.001**	0.72 (0.33–1.11)	**<0.001**
BUN, U/L	10.12 (7.34–12.89)	**<0.001**	7.47 (5.06–9.89)	**<0.001**
Glucose, mmol/L	5.57 (1.45–9.69)	**0.008**		
HCY, *μ*mol/L	0.82 (0.33–1.32)	**0.001**		
LPa, mg/dL	−0.04 (−0.06–−0.03)	**<0.001**	−0.01 (−0.03–0)	0.06
TC, mmol/L	0.1 (−3.67–3.87)	0.96		
D-dimer, mg/L	−8.72 (−14.67–−2.77)	**0.004**	0.02 (−5.27–5.3)	0.995
INR	−20.83 (−57.42–15.75)	0.26	−7.61 (−40.08–24.86)	0.65
eGFR, mL/min per 1.73 m^2^	−0.41 (−0.53–−0.28)	**<0.001**	−0.01 (−0.19–0.18)	0.96
Hypertension grades (3 *vs.* 1-2)	15.81 (7.86–23.75)	**<0.001**	11.58 (4.96–18.19)	**0.001**

SUA, serum uric acid; TyG, triglyceride-glucose index; ALB, serum albumin; hs-CRP, high-sensitivity C-reactive protein; LDL-C, low-density lipoprotein cholesterol; TG, triglyceride; HDL-C, high-density lipoprotein cholesterol; ALT, alanine aminotransferase; AST, aspartate aminotransferase; Scr, blood creatinine; BUN, blood urea nitrogen; HCY, homocysteine; LPa, lipoprotein a; TC, total cholesterol; INR, international normalized ratio; and eGFR: estimated glomerular filtration rate. Statistically significant *P*-values in bold for a better follow-up of the results. The bold values mean that these parameters were associated with SUA in patients with primary hypertension (all *P* < 0.05). Furthermore, the multivariate analysis also revealed that the TyG index was positively associated with SUA after adjusting for potential confounding factors.

**Table 4:** Association between the TyG index and HUA in different models.

TyG index	Crude model		Model 1		Model 2		Model 3	
	OR (95% CI)	*P* value	OR (95% CI)	*P* value	OR (95% CI)	*P* value	OR (95% CI)	*P* value
Per 1 unit increase	2.47 (1.99–3.07)	**<0.001**	2.14 (1.71–2.68)	**<0.001**	1.98 (1.55–2.54)	**<0.001**	1.83 (1.40–2.39)	**<0.001**
*Tertiles*								
Q1	1 (ref)		1 (ref)		1 (ref)		1 (ref)	
Q2	1.66 (1.17–2.35)	**0.004**	1.51 (1.07–2.15)	**0.02**	1.48 (1.01–2.17)	**0.045**	1.45 (0.98–2.15)	0.06
Q3	3.31 (2.39–4.58)	**<0.001**	2.73 (1.96–3.8)	**<0.001**	2.60 (1.79–3.77)	**<0.001**	2.40 (1.60–3.60)	**<0.001**
Trend. test	1.84 (1.57–2.16)	**<0.001**	1.67 (1.42–1.97)	**<0.001**	1.63 (1.36–1.96)	**<0.001**	1.56 (1.28–1.91)	**<0.001**

Model 1 was adjusted for age; model 2 was adjusted for age, sex, ALB, ALT, AST, Scr, BUN, d-dimer, INR, eGFR, and hypertension grades; model 3 was adjusted for all covariables in model 2 plus LDL-C, HDL-C, and LPa. The enalapril group is the reference group. TyG, triglyceride-glucose index; HUA, hyperuricemia; ALB, serum albumin; ALT, alanine aminotransferase; AST, aspartate aminotransferase; Scr, blood creatinine; BUN, blood urea nitrogen; INR, international normalized ratio; eGFR, estimated glomerular filtration rate; LDL-C, low-density lipoprotein cholesterol; HDL-C, high-density lipoprotein cholesterol; LPa, lipoprotein a; OR, odds ratio; and CI, confidence interval. Statistically significant *P*-values in bold for a better follow-up of the results. The bold values mean that when the TyG index was assessed as tertiles, the incidence risks of HUA in the 2nd and 3rd tertiles were 1.45 (95% CI: 0.98 -2.15) and 2.40 (95% CI:1.60 -3.60) times greater than that in the lowest tertile (*P* for trend <0.001).

**Table 5:** Effect modification of TyG on HUA in different models, as stratified by grades of hypertension.

	Nonadjusted		Model 1		Model 2		Model 3	
	OR (95% CI)	*P* value	OR (95% CI)	*P* value	OR (95% CI)	*P* value	OR (95% CI)	*P* value
*Grades 1-2 hypertension*								
tTyG	3.19 (2.3–4.44)	**<0.001**	2.92 (2.09–4.08)	**<0.001**	2.79 (1.88–4.15)	**<0.001**	2.22 (1.44–3.42)	**<0.001**
Tertiles								
Q1	1 (ref)		1 (ref)		1 (ref)		1 (ref)	
Q2	2.83 (1.68–4.78)	**<0.001**	2.69 (1.59–4.54)	**<0.001**	2.67 (1.48–4.82)	**0.001**	2.57 (1.40–4.71)	**0.002**
Q3	4.90 (2.95–8.15)	**<0.001**	4.27 (2.55–7.15)	**<0.001**	3.97 (2.17–7.26)	**<0.001**	3.02 (1.57–5.82)	**0.001**
Trend. test	2.12 (1.68–2.68)	**<0.001**	1.98 (1.56–2.51)	**<0.001**	1.88 (1.42–2.5)	**<0.001**	1.63 (1.2–2.22)	**0.002**
*Grade 3 hypertension*								
tTyG	1.98 (1.49–2.64)	**<0.001**	1.60 (1.19–2.17)	**0.002**	1.54 (1.12–2.12)	**0.008**	1.58 (1.11–2.24)	**0.011**
Tertiles								
Q1	1 (ref)		1 (ref)		1 (ref)		1 (ref)	
Q2	1.00 (0.62–1.63)	0.99	0.85 (0.52–1.4)	**0.53**	0.88 (0.52–1.5)	0.65	0.92 (0.53–1.58)	0.76
Q3	2.34 (1.53–3.59)	**<0.001**	1.78 (1.14–2.78)	**0.01**	1.88 (1.15–3.07)	**0.01**	1.99 (1.17–3.39)	**0.01**
Trend. test	1.60 (1.28–1.99)	**<0.001**	1.40 (1.11–1.76)	**0.004**	1.44 (1.12–1.84)	**0.004**	1.48 (1.13–1.94)	**0.004**
*P value * ^*∗*^ * for interaction*								
tTyG			**0.01**		**0.03**		**0.03**	
Tertiles			**0.008**		**0.02**		**0.02**	

Model 1 was adjusted for age; model 2 was adjusted for age, sex, ALB, ALT, AST, Scr, BUN, d-dimer, INR, eGFR, and hypertension grades; model 3 was adjusted for all covariables in model 2 plus LDL-C, HDL-C, and LPa. The enalapril group is the reference group. ^*∗*^*P* value for the interaction test: 2-way interaction of TyG (total TyG and TyG tertiles) and hypertension grades (grades 1-2 hypertension *vs.* grade 3 hypertension) with HUA. tTyG, total triglyceride-glucose index; other abbreviations are listed in Table 3. Statistically significant *P*-values in bold for a better follow-up of the results. The bold values mean that the differences are significant. And in the 1st -3rd tertiles, the TyG index had a strong significant association with HUA in the grades 1 -2 hypertension group compared to that in the grade 3 hypertension group (*P* for interaction <0.05).

**Table 6:** Effect modification of TyG on SUA in different models, as stratified by grades of hypertension.

	Nonadjusted		Model 1		Model 2		Model 3	
	*β* coefficient (95% CI)	*P* value	*β* coefficient (95% CI)	*P* value	*β* coefficient (95% CI)	*P* value	*β* coefficient (95% CI)	*P* value
*Grades 1-2 hypertension*								
tTyG	48.66 (39.3–58.03)	**<0.001**	44.72 (35.15–54.28)	**<0.001**	32.71 (23.9–41.53)	**<0.001**	28.72 (18.91–38.53)	**<0.001**
Tertiles								
Q1	0 (ref)		0 (ref)		0 (ref)		0 (ref)	
Q2	35.22 (22.55–47.9)	**<0.001**	32.79 (20.15–45.43)	**<0.001**	23.56 (12.33–34.79)	**<0.001**	22.01 (10.55–33.46)	**<0.001**
Q3	59.52 (46.52–72.52)	**<0.001**	53.76 (40.52–67)	**<0.001**	37.04 (24.96–49.12)	**<0.001**	30.82 (17.57–44.08)	**<0.001**
Trend. test	29.87 (23.38–36.37)	**<0.001**	27.02 (20.4–33.64)	**<0.001**	18.55 (12.51–24.59)	**<0.001**	15.54 (8.91–22.17)	**<0.001**
*Grade 3 hypentension*								
tTyG	27.78 (18.46–37.09)	**<0.001**	17.46 (7.91–27.01)	**<0.001**	15 (6.38–23.62)	**0.001**	13.46 (3.95–22.97)	**0.006**
Tertiles								
Q1	0 (ref)		0 (ref)		0 (ref)		0 (ref)	
Q2	6.27 (−7.77–20.31)	**0.38**	−0.31 (−14.07–13.45)	**0.97**	2.1 (−10.24–14.44)	**0.74**	1.85 (−10.96–14.67)	**0.78**
Q3	38.44 (24.77–52.1)	**<0.001**	25.28 (11.51–39.05)	**<0.001**	25.11 (12.66–37.55)	**<0.001**	24.02 (10.34–37.69)	**0.001**
Trend. test	19.5 (12.66–26.35)	**<0.001**	12.92 (6.03–19.82)	**<0.001**	12.88 (6.66–19.11)	**<0.001**	12.5 (5.67–19.33)	**<0.001**
*P value * ^*∗*^ * for interaction*								
tTyG			**0.001**		**0.002**		**0.002**	
Tertiles			**0.004**		**0.02**		**0.02**	

Model 1 was adjusted for age; model 2 was adjusted for age, sex, ALB, ALT, AST, Scr, BUN, d-dimer, INR, eGFR, and hypertension grades; model 3 was adjusted for all covariables in model 2 plus LDL-C, HDL-C, and LPa. The enalapril group served as the reference group. ^*∗*^*P* value for the interaction test: 2-way interaction of TyG (total TyG and tertiles) and hypertension grades (grades 1-2 hypertension vs. grade 3 hypertension) with SUA. tTyG, total triglyceride-glucose index; other abbreviations as in Tables 1 and 2. Statistically significant *P*-values in bold for a better follow-up of the results.The bold values mean that the differences are significant. And in the 1st -3rd tertiles, the TyG index had a strong significant association with SUA in the grades 1 -2 hypertension group compared to that in the grade 3 hypertension group (*P* for interaction <0.05).

## Data Availability

The datasets used and/or analyzed during the current study are available from the corresponding author upon reasonable request.
